# The Prognostic Value of the Hemoglobin, Albumin, Lymphocyte, and Platelet (HALP) Score in Lung Cancer: A Systematic Review and Meta-Analysis

**DOI:** 10.3390/jcm14165701

**Published:** 2025-08-12

**Authors:** Min Zhang, Chuangying Xie, Sitong Liu, Hong Fan, Zhenzhen Li, Xiang Tong

**Affiliations:** 1 Department of Pulmonary and Critical Care Medicine, West China Hospital, Sichuan University, Chengdu 610041, China; 18894266493@163.com (M.Z.); 15984980891@163.com (C.X.); liusitong@wchscu.edu.cn (S.L.); fanhong@scu.edu.cn (H.F.); 2 State Key Laboratory of Respiratory Health and Multimorbidity, Chengdu 610041, China; 3 Health Management Center, West China Hospital of Sichuan University, Chengdu 610041, China

**Keywords:** HALP, lung cancer, overall survival, progression-free survival, meta-analysis

## Abstract

**Background**: Lung cancer remains the leading cause of global cancer mortality. The HALP (hemoglobin, albumin, lymphocyte, platelet) score integrates nutritional, immune, and inflammatory status and may offer prognostic value. This meta-analysis evaluates the association between the HALP score and survival outcomes in lung cancer patients. **Methods**: Following PRISMA guidelines, PubMed, Embase, Web of Science, CNKI, Wanfang, and Google Scholar were searched. Inclusion criteria covered observational studies in lung cancer reporting hazard ratios (HRs) for overall survival (OS), progression-free survival (PFS), or disease-free survival (DFS). Study quality was assessed via the Newcastle–Ottawa Scale (NOS). Random-effects models were used to pool HRs (95% confidence intervals [CIs]), with subgroup and sensitivity analyses used to address heterogeneity. **Results**: Fourteen studies (N = 10,182 patients) were included. A high HALP score predicted significantly improved OS in multivariate analysis (HR = 0.56, 95% CI: 0.46–0.69, *p* < 0.001), representing a 44% mortality risk reduction. The results were consistent for surgical (HR = 0.60, CI: 0.43–0.84), advanced (HR = 0.47, CI: 0.32–0.69), and all-stage subgroups. High HALP also correlated with superior PFS (multivariate HR = 0.56, CI: 0.39–0.78, *p* = 0.001) but not DFS (HR = 0.50, CI: 0.22–1.16, *p* = 0.107). Significant heterogeneity persisted (I^2^ > 75% for OS), likely due to stage variability and non-standard HALP cutoffs. Publication bias was detected for OS studies (Egger′s *p* = 0.003). **Conclusions**: The HALP score is a low-cost, accessible prognostic biomarker for lung cancer. A high HALP score independently predicts better OS and PFS but not DFS, suggesting utility for long-term risk stratification. Standardized HALP thresholds and validation in diverse populations are needed for clinical implementation.

## 1. Introduction

Globally, lung cancer ranks as the second most common malignancy and the leading cause of cancer-related mortality [1]. Globally, 1.8 million fatalities were attributed to pulmonary malignancies in 2020, constituting nearly one-fifth of total cancer-related mortality [2]. Based on histopathological classification, lung cancer can be categorized into two main types: non-small cell lung cancer (NSCLC) and small cell lung cancer (SCLC). NSCLC predominates, constituting approximately 85% of all lung cancer cases [3]. Despite the widespread adoption of the Tumor-Node-Metastasis (TNM) classification as the primary criterion for directing therapeutic strategies and estimating survival outcomes in lung carcinoma [4], significant heterogeneity in clinical outcomes persists among patients at the same disease stage. Therefore, developing prognostic predictors in clinical practice that are simpler, more reliable, and more cost-effective to aid in prognosis assessment is critically important.

The occurrence, progression, and metastasis of lung cancer are closely linked to the host’s nutritional status, immune response, and inflammatory reactions [5,6]. The HALP (hemoglobin, albumin, lymphocyte, and platelet) score, a composite index calculated as (hemoglobin [g/L] × albumin [g/L] × lymphocytes [×10^9^/L])/platelets [×10^9^/L], has been proposed as a novel biomarker reflecting systemic inflammation and nutritional status [7]. Multiple studies have demonstrated that the HALP score can indicate tumor progression and significantly correlates with cancer patients’ long-term clinical outcomes [8,9].

In recent years, emerging evidence has linked the HALP score to prognostic outcomes in lung cancer. Liu et al. demonstrated that individuals stratified by high HALP values showed a pronounced increase in 5-year overall survival (OS) rates relative to their counterparts scoring below the threshold [10]. Wei et al. reported that the HALP score may serve as a prognostic indicator for both OS and disease-free survival (DFS) in NSCLC patients receiving adjuvant chemotherapy [11]. However, conflicting evidence exists regarding the prognostic utility of the HALP score in NSCLC [12]. To date, no systematic review or meta-analysis has comprehensively evaluated the prognostic value of the HALP score across lung cancer subtypes. Therefore, this study aims to undertake the first comprehensive meta-analysis to generate robust evidence-based conclusions regarding the prognostic utility of the HALP score, thereby guiding its rational application in clinical practice.

## 2. Materials and Methods

### 2.1. Data Sources and Search Strings

This study employed a structured literature retrieval strategy aligned with the Preferred Reporting Items for Systematic Reviews and Meta-Analyses (PRISMA) standards to synthesize evidence on the HALP score’s role in lung oncology. As this meta-analysis synthesized observational prognostic data and was not prospectively designed to inform clinical guidelines, PROSPERO registration was not formally required at the time of study conception. The search was performed across the databases PubMed, Web of Science, Embase, CNKI, and Wanfang Database on 1 May 2025. The databases were filtered to include publications in English or Chinese. Studies were identified using search terms related to lung cancer (“lung cancer”, “non-small cell lung cancer”, “small cell lung cancer”, “NSCLC”, “SCLC”) AND HALP (“HALP score”, “HALP index”, “hemoglobin albumin lymphocyte platelet score”, “hemoglobin albumin lymphocyte platelet index”). A supplementary search was performed using Google Scholar with identical search terms. As this meta-analysis exclusively utilized aggregated data derived from previously published studies, neither ethical approval nor patient consent was required.

### 2.2. Inclusion and Exclusion Criteria

Studies meeting the following inclusion criteria were selected as follows: (1) original research involving human subjects; (2) patients with a pathologically confirmed lung cancer diagnosis; and (3) studies evaluating the HALP score as a prognostic factor and reporting sufficient extractable data for meta-analysis. Studies were excluded according to the following criteria: (1) publications in languages other than English or Chinese; (2) reviews, meta-analyses, comments, guidelines, editorials, or letters to the editor; (3) studies reporting duplicate datasets; and (4) insufficient extractable data for quantitative synthesis (meta-analysis). In cases where two publications overlapped either entirely or partially concerning the same study population, the publication reporting data on the largest cohort was included.

### 2.3. Study Quality Score Evaluation

The methodological quality of the identified studies investigating the role of the HALP score in lung cancer was assessed using the Newcastle–Ottawa Scale (NOS). The NOS evaluates quality across three domains: selection, comparability, and exposure. Total NOS scores range from 0 to 9, with scores of 0–3, 4–6, and 7–9 indicating low, moderate, and high quality, respectively [13]. Quality assessment was performed through a consensus process involving all authors.

### 2.4. Data Extraction

According to the predefined inclusion and exclusion criteria, two independent authors (Zhang M and Xie CY) systematically extracted demographic information and parameters from each primary study using a standardized data extraction template in Microsoft Excel^®^. Discrepancies in extracted data were resolved through consensus-based discussion. The extraction protocol encompassed the following variables: first author, year of publication, country of origin, participant age, sample size, Receiver Operating Characteristic-determined cut-off value for the HALF score, and hazard ratios (HRs) with corresponding 95% confidence intervals (CIs) for OS, DFS, and progression-free survival (PFS). The reference management software EndNote^®^ (Clarivate Analytics, Version X9) was employed for the identification and removal of duplicate records during the screening phase.

### 2.5. Statistical Methods

All statistical analyses were performed using STATA 14.0 (StataCorp LP, College Station, TX, USA). Where appropriate, HRs were converted to logarithmic scales prior to synthesis. A random-effects model was employed for meta-analytical pooling based on anticipated methodological heterogeneity. This approach produces more conservative effect estimates with wider confidence intervals than fixed-effect models while reducing false-positive (Type I) error rates under conditions of significant inter-study variation [14]. We employed meta-regression, subgroup analysis, and sensitivity analysis to assess potential sources of heterogeneity. Effect estimates included HRs with corresponding 95%CIs reflecting the association between HALP scores and OS, DFS, and PFS. Between-study heterogeneity was quantified using Cochran’s Q statistic (with χ^2^ distribution) and the I^2^ metric. Substantial heterogeneity was defined a priori as either (1) a Cochran’s Q test *p*-value < 0.10 or (2) an I^2^ value > 50%.

## 3. Results

### 3.1. Study Characteristics

**Figure 1:**
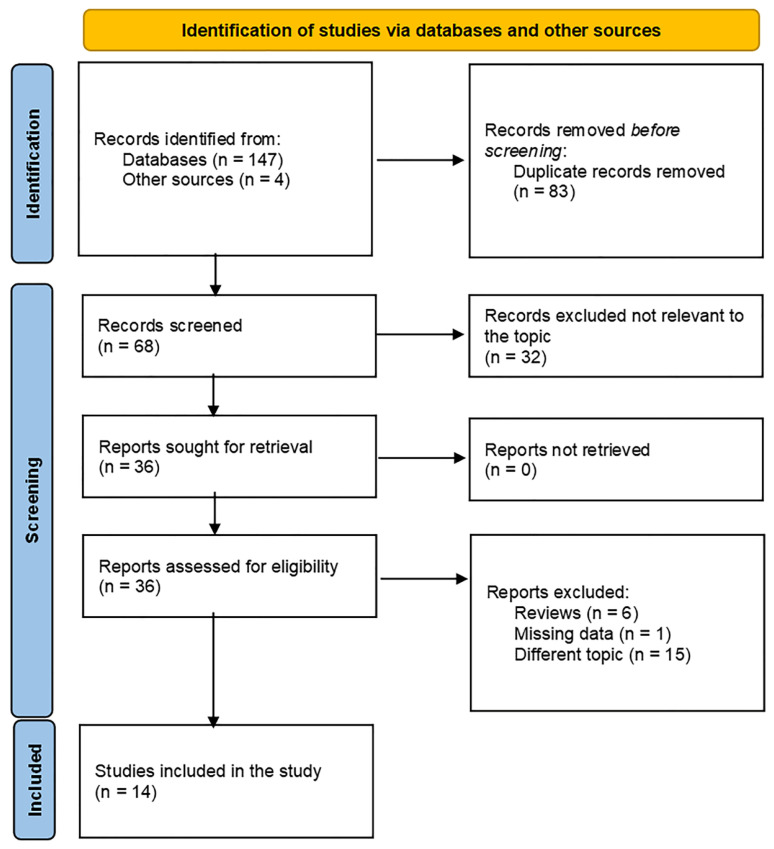
The flow diagram of included and excluded studies.

A total of 151 articles were identified through the initial searches of PubMed, Embase, Web of Science, CNKI, Wanfang Database, and Google Scholar. As depicted in the flow diagram (Figure 1), 83 duplicate records were excluded. Following title and abstract screening, 32 articles were excluded for not investigating the association between the HALP score and lung cancer. The remaining 36 articles underwent full-text assessment. Of these, 22 were excluded for the following reasons: being review articles (n = 6), not evaluating the prognostic value of the HALP score (n = 15), or providing no extractable data (n = 1). Consequently, 14 eligible articles, including 10,182 patients, were ultimately included in the current meta-analysis [10,11,12,15,16,17,18,19,20,21,22,23,24,25] (Table 1).

Among the included studies, only one study enrolled patients with SCLC [23]; the remaining studies focused on NSCLC [10,11,12,15,16,17,18,19,20,21,22,23,24,25]. Ten studies (71.4%) were conducted in China [10,11,15,18,19,21,22,23,24,25], two (14.3%) in Turkey [16,20], and one each in the UK [12] and France [17]. Regarding patient populations, six studies (42.9%) included patients who underwent surgical resection [11,12,15,17,21,24], five (35.7%) included patients with locally advanced or metastatic disease [16,18,19,20,22], and three (21.4%) encompassed patients across all disease stages [10,23,25]. Thirteen studies (92.9%) reported HRs for OS, five (35.7%) reported HRs for PFS, and only two (14.3%) reported HRs for DFS (Table 1). In all the studies, the HALP score was derived from patients before treatment or surgery. According to the Newcastle–Ottawa Scale (NOS) quality assessment, one included study (7.1%) was rated as having moderate quality, while the other thirteen studies (92.9%) were deemed high quality (Table 2).

### 3.2. HLAP Score and OS

**Figure 2:**
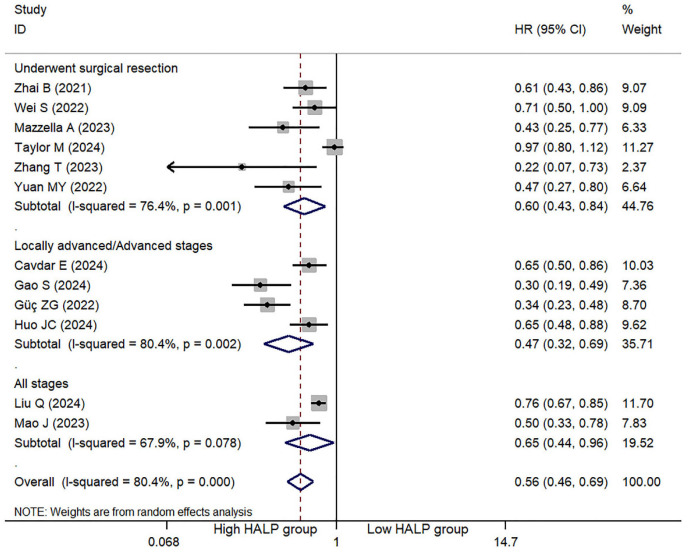
Multivariate analysis of the association between the HALP score and overall survival in lung cancer patients [10,11,12,15,16,17,19,20,21,22,24,25]. The arrow indicates that one or both ends of its confidence interval have been truncated. The diamond marker represents the pooled effect estimate and its 95% confidence interval.

Eleven studies reported HRs from univariate analyses examining the association between the HALP score and OS, while twelve studies reported HRs from multivariate analyses (Table 3). The meta-analysis demonstrated that a high HALP score was significantly associated with a decreased risk of death in patients with lung cancer, specifically showing a 49% reduced risk of mortality in the univariate analysis (HR: 0.51, 95% CI = 0.41–0.62, *p* < 0.001) (Supplementary Figure S1) and a 44% reduced risk in the multivariate analysis (HR: 0.56, 95% CI = 0.46–0.69, *p* < 0.001) (Figure 2); substantial between-study heterogeneity was observed in these analyses (I^2^ = 77.6% for univariate, I^2^ = 80.4% for multivariate). The meta-regression analysis did not reveal significant effects of publication year, country of origin, or tumor stage on heterogeneity (*p* = 0.852, 0.495, 0.840, respectively).

Furthermore, subgroup analyses were performed, consistently demonstrating a significant association between high HALP score and reduced mortality risk: for patients undergoing surgical resection, the univariate HR was 0.45 (95% CI = 0.30–0.68, *p* < 0.001) and the multivariate HR was 0.60 (95% CI = 0.43–0.84, *p* = 0.003); for patients with locally advanced or metastatic disease, the univariate HR was 0.55 (95% CI = 0.39–0.78, *p* = 0.001) (Supplementary Figure S1) and the multivariate HR was 0.47 (95% CI = 0.32–0.69, *p* < 0.001) (Figure 2); and for studies encompassing patients across all disease stages, the univariate HR was 0.47 (95% CI = 0.26–0.82, *p* = 0.008) and the multivariate HR was 0.65 (95% CI = 0.44–0.96, *p* = 0.029). Nevertheless, significant heterogeneity persisted within each subgroup, likely reflecting substantial variations in the disease stage distribution among the included studies.

### 3.3. HLAP Score and PFS/DFS

**Figure 3:**
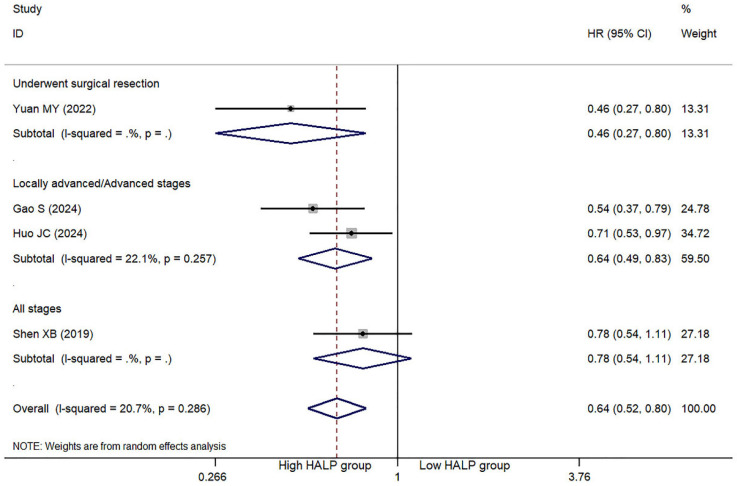
Multivariate analysis of the association between the HALP score and progression-free survival in lung cancer patients [19,22,23,24]. The diamond marker represents the pooled effect estimate and its 95% confidence interval.

This meta-analysis included five studies evaluating the association between the HALP score and PFS in lung cancer (Table 3). Both univariate and multivariate analyses demonstrated that a high HALP score was significantly associated with a reduced risk of PFS in the overall analysis (univariate: HR = 0.67, 95% CI = 0.56–0.80, *p* < 0.001) (Supplementary Figure S2); multivariate: HR = 0.64, 95% CI = 0.39–0.78, *p* = 0.001 (Figure 3). However, the subgroup analysis focusing on patients across all disease stages (n = 1 study) showed no significant association between a high HALP score and a reduced PFS risk in the multivariate analysis (HR = 0.78, 95% CI = 0.54–1.11, *p* = 0.167) (Figure 3). Additionally, two studies investigated the association between the HALP score and DFS in lung cancer. The results indicated that no significant association in either the univariate (HR = 0.31, 95% CI = 0.06–1.52, *p* = 0.148) or multivariate analysis (HR = 0.50, 95% CI = 0.22–1.16, *p* = 0.107) (Supplementary Figure S3).

### 3.4. Sensitivity Analysis and Publication Bias

**Figure 4:**
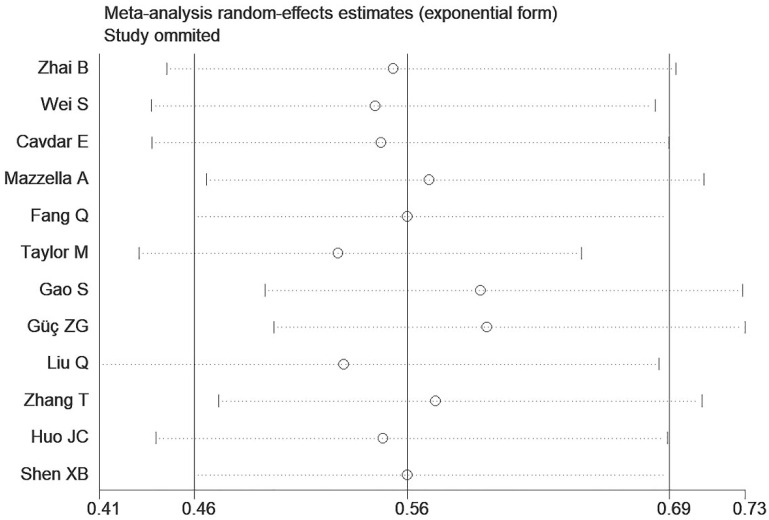
Sensitivity analysis of the association between the HALP score and overall survival in lung cancer patients.

Substantial heterogeneity was observed across both the overall and subgroup analyses within the present study. Consequently, a sensitivity analysis was performed; the results demonstrated that the overall estimates remained statistically significant and stable following sequential exclusion of individual studies, indicating reliable findings despite the substantial heterogeneity (Figure 4). Furthermore, a publication bias assessment was conducted for studies investigating the association between the HALP score and OS in lung cancer. Funnel plot inspection revealed substantial publication bias (Supplementary Figure S4), which was statistically confirmed by both Begg’s test (*p* = 0.005) and Egger’s test (*p* = 0.003).

## 4. Discussion

This meta-analysis provides the first comprehensive synthesis of evidence establishing the HALP score as a significant prognostic biomarker in lung cancer. The key finding was that patients with a higher HALP demonstrated a 44% reduction in mortality risk, with consistent effects observed across surgical, advanced, and all-stage subgroups. Additionally, this study found that a higher HALP score was significantly associated with better PFS in lung cancer patients, but it was not associated with DFS. However, due to the exploratory nature of the DFS analysis resulting from limited data, overinterpretation should be avoided.

Previous studies have established that tumor-related inflammation and nutritional–immunological imbalance constitute significant foundational factors in cancer initiation, progression, and metastasis [26,27,28,29,30]. Anemia is observed in approximately 40% to 64% of patients seeking cancer treatment, and its presence correlates with both diminished quality of life and reduced survival duration [31]. It is hypothesized that tumoral hypoxia may contribute to tumor growth and treatment resistance by promoting angiogenesis, inducing genetic mutations, fostering apoptosis resistance, and conferring resistance to free radicals generated by chemotherapy and radiotherapy [31,32,33]. In cancer patients, serum albumin remains clinically indispensable for assessing nutritional status, disease severity, progression, and prognosis. Notably, serum albumin levels have been identified as an independent prognostic factor for survival across multiple cancer types [34,35]. Concurrently, lymphocytopenia and thrombocytosis have been substantiated as closely associated with tumorigenesis, potentially mediated through mechanisms involving immune evasion, enhanced tumor angiogenesis, and metastasis [36,37].

The HALP score, calculated from four routine hematological parameters (hemoglobin, albumin, lymphocyte, and platelet), comprehensively reflects the host’s integrated nutritional status, immunological competence, and inflammatory state. This multifaceted representation elucidates its robust association [26,27,28,29,30] with survival outcomes. In this study, patients in the high-HALP group demonstrated a significantly reduced risk of all-cause mortality, underscoring HALP’s promise as an integrated physiological status predictor for lung cancer prognosis. This finding aligns with research on gastric, colorectal, and other malignancies [38,39], further supporting HALP’s potential as a versatile prognostic indicator. Furthermore, though limited to only two reported studies, HALP failed to demonstrate prognostic value for DFS [11,21], warranting further investigation in future research. Notably, this observation suggests that HALP may prove more clinically useful for predicting long-term survival outcomes than early disease recurrence.

The TNM staging system primarily reflects the anatomical extent of tumor invasion, yet significant survival heterogeneity persists among patients within the same stage. The HALP score provides a low-cost, repeatable tool that detects tumor microenvironment alterations earlier than imaging. Incorporating HALP into the TNM framework addresses these limitations by identifying high-risk subgroups within the same stage, enhancing prognostic stratification accuracy, and optimizing individualized therapy [7,40]. For example, patients with low HALP scores may require prioritized nutritional or anti-inflammatory interventions to improve survival outcomes. Furthermore, HALP quantifies multidimensional pathophysiological interactions (nutritional, immunological, coagulative, and inflammatory) more comprehensively than indices like the Glasgow Prognostic Score (GPS) or the Prognostic Nutritional Index (PNI). Its robustness against confounding factors—such as concurrent infections, systemic inflammation, or metabolic complications—outperforms the GPS and PNI, which are frequently skewed in these clinical scenarios [41,42], underscoring HALP’s value as a comprehensive biomarker in oncology.

However, substantial heterogeneity persisted despite rigorous subgroup analyses. This likely stems from significant variations in disease stages across the included studies. For instance, six studies exclusively enrolled early/intermediate-stage lung cancer patients eligible for surgical resection without distant metastasis, whereas five others solely recruited advanced-stage patients with metastatic disease. Sex differences, age distribution, and study design could contribute to heterogeneity; however, these factors were not analyzed because of insufficient data extractability in this study. Although sensitivity analyses confirmed result stability, supporting the robustness of our primary conclusions, these findings require cautious interpretation. Furthermore, significant publication bias and geographic skew (71.4% Chinese cohorts) constrain generalizability, potentially explaining part of the observed heterogeneity. Prospective validation in Western populations is urgently needed to address sampling bias. Crucially, the lack of standardized HALP cut-off values across studies may substantially contribute to heterogeneity. Future prospective studies must validate and standardize these thresholds to enhance clinical utility.

Although this meta-analysis demonstrates substantial representativeness and robust methodological quality, several limitations warrant acknowledgment. First, the predominance of retrospective cohort studies among the included investigations introduces potential selection bias. Second, our search strategy neither incorporated unpublished studies nor solicited missing data from original authors—an approach that may elevate publication bias risk. Third, while some studies report loss of HALP’s prognostic significance in patients > 65 years [10], we were unable to conduct robust subgroup stratification by age, tumor stage, or treatment regimens due to insufficient data granularity.

## 5. Conclusions

This meta-analysis confirms that the HALP score is a valuable, affordable, and accessible predictor of lung cancer outcomes. Patients with higher HALP scores exhibited a 44% lower risk of death and longer PFS. However, HALP did not predict DFS, suggesting it may be more effective for predicting long-term survival than detecting early recurrence. Future studies are needed to establish standardized HALP thresholds and confirm its utility, particularly in Western patient populations.

## Figures and Tables

**Table 1:** Characteristics of the studies on the correlation between the HALP score and lung cancer.

First Author	Year	Country	N	M/F	Age	Type	Classification	Cut-off	High HALP	Low HALP	Outcome
Zhai B [15]	2021	China	238	150/80	62.3 ± 8.4	NSCLC	Underwent surgical resection	48	139	99	OS
Wei S [11]	2022	China	362	217/145	NA	NSCLC	Underwent surgical resection	48.2	127	77	OS, DFS
Cavdar E [16]	2024	Turkey	278	260/18	40-82	NSCLC	Locally advanced/Advanced stages	26	139	139	OS
Mazzella A [17]	2023	France	257	149/108	65 ± 10.2	NSCLC	Underwent surgical resection	32.2	91	66	OS
Fang Q [18]	2023	China	223	189/34	60.4	NSCLC	Locally advanced/Advanced stages	39.33	111	112	OS, PFS
Taylor M [12]	2024	UK	5029	2444/2585	68.6 ± 9.1	NSCLC	Underwent surgical resection	36.87			OS
Gao S [19]	2024	China	203	140/63	59.6 ± 9.7	NSCLC	Locally advanced/Advanced stages	28.02	71	132	OS, PFS
Güç ZG [20]	2022	Turkey	401	317/84	63.47 ± 9.75	NSCLC	Locally advanced/Advanced stages	23.24	171	230	OS
Liu Q [10]	2024	China	2053	1346/707	60.73 ± 9.8	NSCLC	All stages	29.17	1090	963	OS
Zhang T [21]	2023	China	52	35/17	43-79	NSCLC	Underwent surgical resection	24.3	46	6	OS, DFS
Huo JC [22]	2024	China	206	193/13	28-83	NSCLC	Locally advanced/Advanced stages	24.3	127	79	OS, PFS
Shen XB [23]	2019	China	178	142/36	61.24 ± 9.27	SCLC	All stages	25.8	130	48	PFS
Yuan MY [24]	2022	China	270	166/104	68 ± 6	NSCLC	Underwent surgical resection	39	104	166	OS, PFS
Mao J [25]	2023	China	432	256/176	NA	NSCLC	All stages	42.2	185	247	OS

N, number of patients; M/F, male/female; NA, not applicable; NSCLC, non-small cell lung cancer; SCLC, small cell lung cancer; Cut-off, the cutoff value of HALF; OS, overall survival; DFS, disease-free survival; PFS, progression-free survival.

**Table 2:** Quality assessment of the included studies using the Newcastle–Ottawa scale.

Author	Selection				Comparability		Exposure			Total
	1. Representativeness of Exposed Cohorts	2. Selection of Non-Exposed Cohorts	3. Ascertainment of Exposure	4. Demonstration that Outcome of Interest was not Present at Start of Study	1. Study Controls for the Most Important Factor	2. Comparability for Any Additional Factor	1. Assessment of Outcome	2. Was Follow-Up Long Enough for Outcomes to Occur	3. Adequacy of Follow-Up	
Zhai B [15]	*	*	*	*	-	*	*	*	*	8
Wei S [11]	*	*	*	*	*	-	*	*	*	8
Cavdar E [16]	*	*	*	*	-	*	*	*	-	7
Mazzella A [17]	*	*	*	*	*	*	*	*	-	8
Fang Q [18]	*	*	*	*	*	*	*	*	-	8
Taylor M [12]	*	*	*	*	-	-	*	*	-	6
Gao S [19]	*	*	*	*	*	-	*	*	*	8
Güç ZG [20]	*	*	*	*	-	-	*	*	*	7
Liu Q [10]	*	*	*	*	*	-	*	*	*	8
Zhang T [21]	*	*	*	*	*	-	*	*	*	8
Huo JC [22]	*	*	*	*	-	*	*	*	*	8
Shen XB [23]	*	*	*	*	*	*	*	*	-	8
Yuan MY [24]	*	*	*	*	-	-	*	*	*	7
Mao J [25]	*	*	*	*	*	-	*	*	*	8

Asterisk means that satisfaction is based on the NOS.

**Table 3:** Effect sizes extracted from the included studies for meta-analysis.

First Author	OS Univariate			OS Multivariate			DFS Univariate			DFS Multivariate			PFS Univariate			PFS Multivariate		
	HR	LCI	UCI	HR	LCI	UCI	HR	LCI	UCI	HR	LCI	UCI	HR	LCI	UCI	HR	LCI	UCI
Zhai B [15]	0.531	0.379	0.744	0.610	0.433	0.860	/	/	/	/	/	/	/	/	/	/	/	/
Wei S [11]	0.672	0.479	0.942	0.707	0.503	0.995	0.648	0.475	0.882	0.671	0.491	0.916	/	/	/	/	/	/
Cavdar E [16]	0.630	0.480	0.820	0.650	0.500	0.860	/	/	/	/	/	/	/	/	/	/	/	/
Mazzella A [17]	0.360	0.210	0.610	0.430	0.250	0.770	/	/	/	/	/	/	/	/	/	/	/	/
Fang Q [18]	0.996	0.709	1.401	/	/	/	/	/	/	/	/	/	0.771	0.569	1.045	/	/	/
Taylor M [12]	/	/	/	0.975	0.800	1.118	/	/	/	/	/	/	/	/	/	/	/	/
Gao S [19]	0.380	0.240	0.600	0.300	0.190	0.490	/	/	/	/	/	/	0.600	0.420	0.850	0.540	0.370	0.790
Güç ZG [20]	0.333	0.230	0.482	0.335	0.231	0.484	/	/	/	/	/	/	/	/	/	/	/	/
Liu Q [10]	0.613	0.546	0.690	0.756	0.671	0.853	/	/	/	/	/	/	/	/	/	/	/	/
Zhang T [21]	0.172	0.066	0.443	0.224	0.068	0.733	0.126	0.046	0.347	0.268	0.085	0.847	/	/	/	/	/	/
Huo JC [22]	0.589	0.437	0.793	0.650	0.481	0.880	/	/	/	/	/	/	0.631	0.469	0.850	0.715	0.528	0.968
Shen XB [23]	/	/	/	/	/	/	/	/	/	/	/	/	/	/	/	0.777	0.544	1.112
Yuan MY [24]	/	/	/	0.466	0.273	0.795	/	/	/	/	/	/	/	/	/	0.460	0.266	0.796
Mao J [25]	0.343	0.233	0.423	0.503	0.325	0.778	/	/	/	/	/	/	/	/	/	/	/	/

OS, overall survival; DFS, disease-free survival; PFS, progression-free survival; HR, hazard ratio; LCI, lower confidence interval; UCI, upper confidence interval.

## Data Availability

The original contributions presented in this study are included in this article/the Supplementary Materials. Further inquiries can be directed to the corresponding authors.

## Supplementary Materials

The following supporting information can be downloaded at: https://www.mdpi.com/article/10.3390/jcm14165701/s1, Figure S1: Univariate analysis of the association between HALP score and overall survival in lung cancer patients. Figure S2: Univariate analysis of the association between HALP score and progression-free survival in lung cancer patients. Figure S3: Multivariate analysis of the association between HALP score and disease-free survival in lung cancer patients. Figure S4:Funnel plot analysis for the association between HALP score and overall survival in lung cancer patients.

## Abbreviations

HALP	Hemoglobin, Albumin, Lymphocyte, and Platelet
NSCLC	Non-Small Cell Lung Cancer
SCLC	Small Cell Lung Cancer
TNM	Tumor, Node, Metastasis (staging system)
OS	Overall Survival
DFS	Disease-Free Survival
PRISMA	Preferred Reporting Items for Systematic Reviews and Meta-Analyses
CNKI	China National Knowledge Infrastructure
HR	Hazard Ratio
CI	Confidence Interval
PFS	Progression-Free Survival
NOS	Newcastle–Ottawa Scale

## References

[1] Sharma R. (2022). Mapping of global, regional and national incidence, mortality and mortality-to-incidence ratio of lung cancer in 2020 and 2050. Int. J. Clin. Oncol..

[2] Sung H., Ferlay J., Siegel R.L., Laversanne M., Soerjomataram I., Jemal A., Bray F. (2021). Global Cancer Statistics 2020: GLOBOCAN Estimates of Incidence and Mortality Worldwide for 36 Cancers in 185 Countries. CA Cancer J. Clin..

[3] Ettinger D.S., Akerley W., Bepler G., Blum M.G., Chang A., Cheney R.T., Chirieac L.R., D’Amico T.A., Demmy T.L., Ganti A.K.P. (2010). Non-small cell lung cancer. J. Natl. Compr. Cancer Netw..

[4] Rami-Porta R., Nishimura K.K., Giroux D.J., Detterbeck F., Cardillo G., Edwards J.G., Fong K.M., Giuliani M., Huang J., Kernstine K.H. (2024). The International Association for the Study of Lung Cancer Lung Cancer Staging Project: Proposals for Revision of the TNM Stage Groups in the Forthcoming (Ninth) Edition of the TNM Classification for Lung Cancer. J. Thorac. Oncol. Off. Publ. Int. Assoc. Study Lung Cancer.

[5] Liu C.A., Liu T., Li H.C., Song M.M., Ge Y.Z., Ruan G.T., Deng L., Zhang Q., Xie H.-L., Lin S.-Q. (2023). Nutrition impact symptoms: Noteworthy prognostic indicators for lung cancer. Clin. Nutr..

[6] Xie H., Ruan G., Ge Y., Zhang Q., Zhang H., Lin S., Song M., Zhang X., Liu X., Li X. (2022). Inflammatory burden as a prognostic biomarker for cancer. Clin. Nutr..

[7] Chen X.L., Xue L., Wang W., Chen H.N., Zhang W.H., Liu K., Chen X.-Z., Yang K., Zhang B., Chen Z.-X. (2015). Prognostic significance of the combination of preoperative hemoglobin, albumin, lymphocyte and platelet in patients with gastric carcinoma: A retrospective cohort study. Oncotarget.

[8] Feng J.F., Wang L., Yang X. (2021). The preoperative hemoglobin, albumin, lymphocyte and platelet (HALP) score is a useful predictor in patients with resectable esophageal squamous cell carcinoma. Bosn. J. Basic Med. Sci..

[9] Yalav O., Topal U., Unal A.G., Eray I.C. (2021). Prognostic significance of preoperative hemoglobin and albumin levels and lymphocyte and platelet counts (HALP) in patients undergoing curative resection for colorectal cancer. Ann. Ital. Chir..

[10] Liu Q., Xie H., Cheng W., Liu T., Liu C., Zhang H., Lin S., Liu X., Tian H., Li X. (2024). The preoperative hemoglobin, albumin, lymphocyte, and platelet score (HALP) as a prognostic indicator in patients with non-small cell lung cancer. Front. Nutr..

[11] Wei S., Shao J., Wang J., Wang G. (2022). The preoperative hemoglobin, albumin, lymphocyte, and platelet score is a prognostic factor for non-small cell lung cancer patients undergoing adjuvant chemotherapy: A retrospective study. Ann. Transl. Med..

[12] Taylor M., Evison M., Michael S., Obale E., Fritsch N.C., Abah U., Smith M., Martin G.P., Shackcloth M., Granato F. (2024). Pre-Operative Measures of Systemic Inflammation Predict Survival After Surgery for Primary Lung Cancer. Clin. Lung Cancer.

[13] Stang A. (2010). Critical evaluation of the Newcastle-Ottawa scale for the assessment of the quality of nonrandomized studies in meta-analyses. Eur. J. Epidemiol..

[14] Borenstein M., Hedges L., Rothstein H. (2007). Meta-Analysis: Fixed Effect vs. Random Effects.

[15] Zhai B., Chen J., Wu J., Yang L., Guo X., Shao J., Xu H., Shen A. (2021). Predictive value of the hemoglobin, albumin, lymphocyte, and platelet (HALP) score and lymphocyte-to-monocyte ratio (LMR) in patients with non-small cell lung cancer after radical lung cancer surgery. Ann. Transl. Med..

[16] Cavdar E., Karaboyun K., Kara K. (2024). Comprehensive analysis of the prognostic role of laboratory indices in advanced lung cancer patients. Asia-Pac. J. Clin. Oncol..

[17] Mazzella A., Maiolino E., Maisonneuve P., Loi M., Alifano M. (2023). Systemic Inflammation and Lung Cancer: Is It a Real Paradigm? Prognostic Value of Inflammatory Indexes in Patients with Resected Non-Small-Cell Lung Cancer. Cancers.

[18] Fang Q., Yu J., Li W., Luo J., Deng Q., Chen B., He Y., Zhang J., Zhou C. (2023). Prognostic value of inflammatory and nutritional indexes among advanced NSCLC patients receiving PD-1 inhibitor therapy. Clin. Exp. Pharmacol. Physiol..

[19] Gao S., Huang Q., Wei S., Lv Y., Xie Y., Hao Y. (2024). Prognostic nomogram based on pre-treatment HALP score for patients with advanced non-small cell lung cancer. Clinics.

[20] Güç Z.G., Alacacıoğlu A., Kalender M.E., Oflazoğlu U., Ünal S., Yıldız Y., Salman T., Küçükzeybek Y., Tarhan M.O. (2022). HALP score and GNRI: Simple and easily accessible indexes for predicting prognosis in advanced stage NSCLC patients. The İzmir oncology group (IZOG) study. Front. Nutr..

[21] Zhang T., Liu W., Xu C. (2023). Correlation analysis of hemoglobin, albumin, lymphocyte, platelet score and platelet to albumin ratio and prognosis in patients with lung adenosquamous carcinoma. Front. Oncol..

[22] Huo J.C., Wang Y., Su J.W., Liu S., Osoegawa A., Jia Z.F., Wang Y.-X., Yang J. (2024). Correlation of hemoglobin, albumin, lymphocyte, and platelet score with prognosis in patients with stage III squamous lung cancer. J. Thorac. Dis..

[23] Shen X.B., Zhang Y.X., Wang W., Pan Y.Y. (2019). The Hemoglobin, Albumin, Lymphocyte, and Platelet (HALP) Score in Patients with Small Cell Lung Cancer Before First-Line Treatment with Etoposide and Progression-Free Survival. Med. Sci. Monit. Int. Med. J. Exp. Clin. Res..

[24] Yuan M., Wang P., Meng R., Yang J., Zhou C., Jiao Z. (2022). Predictive value of preoperative hemoglobin, albumin, lymphocyte and platelet (HALP) score for prognosis in elderly patients with non-small cell lung cancer undergoing surgery. Pract. Geriatr..

[25] Mao J., Ji S., Zhang Z., Zhang H. (2023). Prognostic value of a hemoglobin-albumin-lymphocyte-platelet nomogram in non-small cell lung cancer. Chin. Clin. Dr..

[26] Garner H., de Visser K.E. (2020). Immune crosstalk in cancer progression and metastatic spread: A complex conversation. Nat. Rev. Immunol..

[27] Wiseman M.J. (2019). Nutrition and cancer: Prevention and survival. Br. J. Nutr..

[28] Saha S.K., Lee S.B., Won J., Choi H.Y., Kim K., Yang G.M., Dayem A.A., Cho S.-G. (2017). Correlation between Oxidative Stress, Nutrition, and Cancer Initiation. Int. J. Mol. Sci..

[29] Sarvaiya P.J., Guo D., Ulasov I., Gabikian P., Lesniak M.S. (2013). Chemokines in tumor progression and metastasis. Oncotarget.

[30] Gonda T.A., Tu S., Wang T.C. (2009). Chronic inflammation, the tumor microenvironment and carcinogenesis. Cell Cycle.

[31] Varlotto J., Stevenson M.A. (2005). Anemia, tumor hypoxemia, and the cancer patient. Int. J. Radiat. Oncol. Biol. Phys..

[32] Tas F., Eralp Y., Basaran M., Sakar B., Alici S., Argon A., Gulistan B., Hakan C., Adnan A., Erkan T. (2002). Anemia in oncology practice: Relation to diseases and their therapies. Am. J. Clin. Oncol..

[33] Littlewood T.J. (2001). The impact of hemoglobin levels on treatment outcomes in patients with cancer. Semin. Oncol..

[34] Nazha B., Moussaly E., Zaarour M., Weerasinghe C., Azab B. (2015). Hypoalbuminemia in colorectal cancer prognosis: Nutritional marker or inflammatory surrogate?. World J. Gastrointest. Surg..

[35] Almasaudi A.S., Dolan R.D., Edwards C.A., McMillan D.C. (2020). Hypoalbuminemia Reflects Nutritional Risk, Body Composition and Systemic Inflammation and Is Independently Associated with Survival in Patients with Colorectal Cancer. Cancers.

[36] Ikeda M., Furukawa H., Imamura H., Shimizu J., Ishida H., Masutani S., Tatsuta M., Satomi T. (2002). Poor prognosis associated with thrombocytosis in patients with gastric cancer. Ann. Surg. Oncol..

[37] Feng F., Zheng G., Wang Q., Liu S., Liu Z., Xu G., Wang F., Guo M., Lian X., Zhang H. (2018). Low lymphocyte count and high monocyte count predicts poor prognosis of gastric cancer. BMC Gastroenterol..

[38] Sargin Z.G., Dusunceli I. (2022). The Effect of HALP Score on the Prognosis of Gastric Adenocarcinoma. J. Coll. Physicians Surg.—Pak..

[39] Okazaki K., Furukawa K., Haruki K., Onda S., Shirai Y., Tsunematsu M., Taniai T., Matsumoto M., Hamura R., Akaoka M. (2025). Prognostic significance of the hemoglobin, albumin, lymphocyte, platelet (HALP) score after hepatectomy for colorectal liver metastases. Surg. Today.

[40] Farag C.M., Antar R., Akosman S., Ng M., Whalen M.J. (2023). What is hemoglobin, albumin, lymphocyte, platelet (HALP) score? A comprehensive literature review of HALP’s prognostic ability in different cancer types. Oncotarget.

[41] Portale G., Cavallin F., Cipollari C., Spolverato Y., Di Miceli D., Zuin M., Mazzeo A., Morabito A., Sava T., Fiscon V. (2023). Preoperative Prognostic Nutritional Index was not predictive of short-term complications after laparoscopic resection for rectal cancer. Langenbeck’s Arch. Surg..

[42] Aro R., Meriläinen S., Sirniö P., Väyrynen J.P., Pohjanen V.M., Herzig K.H., Rautio T.T., Mäkäräinen E., Häivälä R., Klintrup K. (2022). Sarcopenia and Myosteatosis Are Associated with Neutrophil to Lymphocyte Ratio but Not Glasgow Prognostic Score in Colorectal Cancer Patients. J. Clin. Med..

