# Supplementary material for: The Prognostic Value of the Hemoglobin, Albumin, Lymphocyte, and Platelet (HALP) Score in Lung Cancer: A Systematic Review and Meta-Analysis
Source: J Clin Med. 2025 Aug 12;14(16):5701. doi: 10.3390/jcm14165701 (PMC12386590; doi:10.3390/jcm14165701)
Supplement: Supplementary file 1 [file jcm-14-05701-s001.zip › jcm-3729357-supplementary.pdf]

Supplementary Materials

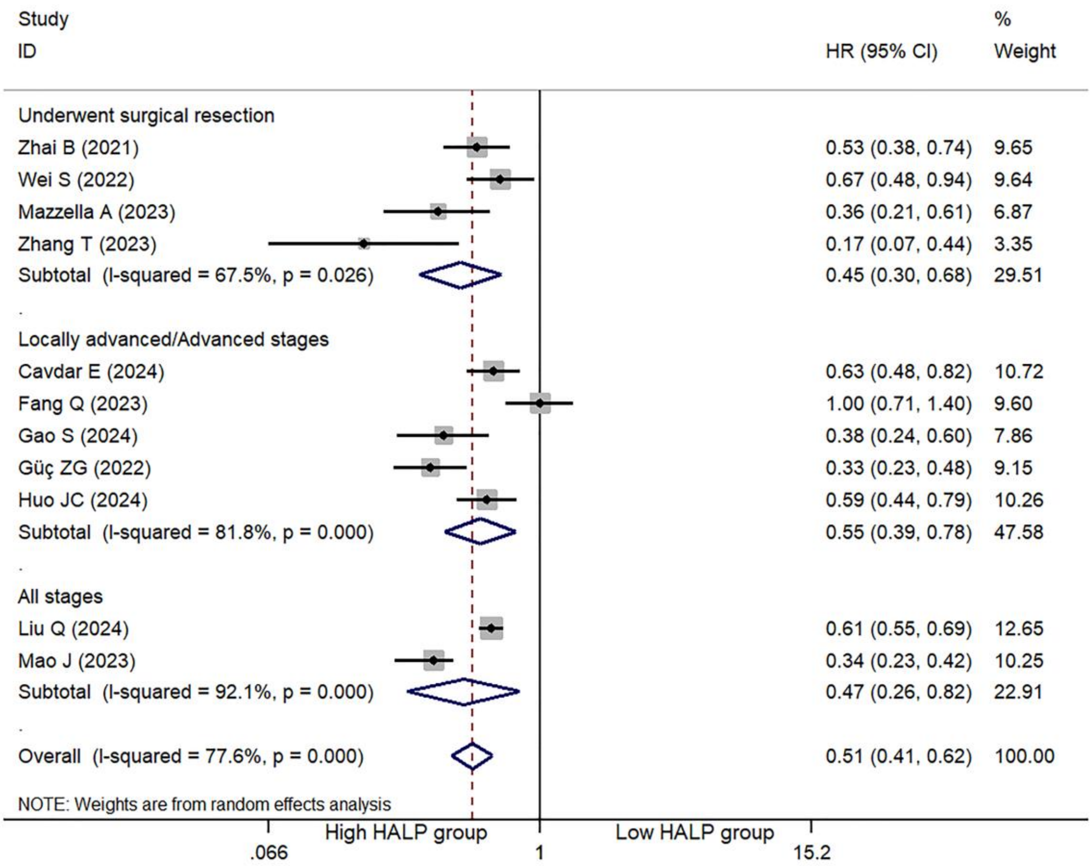

Figure S1: Univariate analysis of the association between HALP score and overall survival in lung cancer patients.

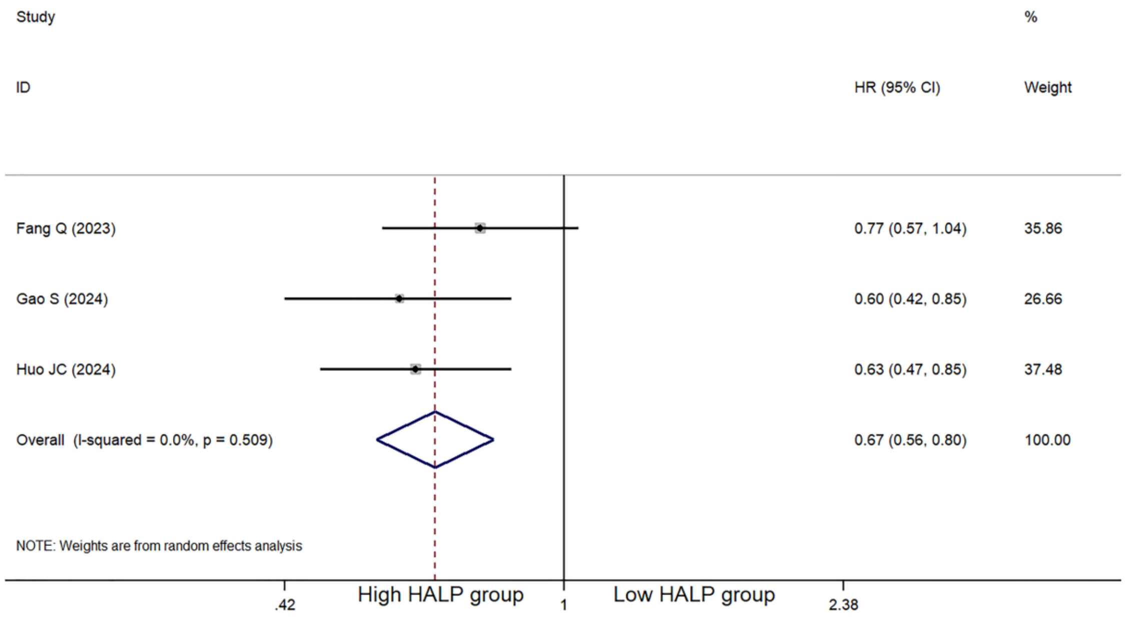

Figure S2: Univariate analysis of the association between HALP score and progression-free survival in lung cancer patients.

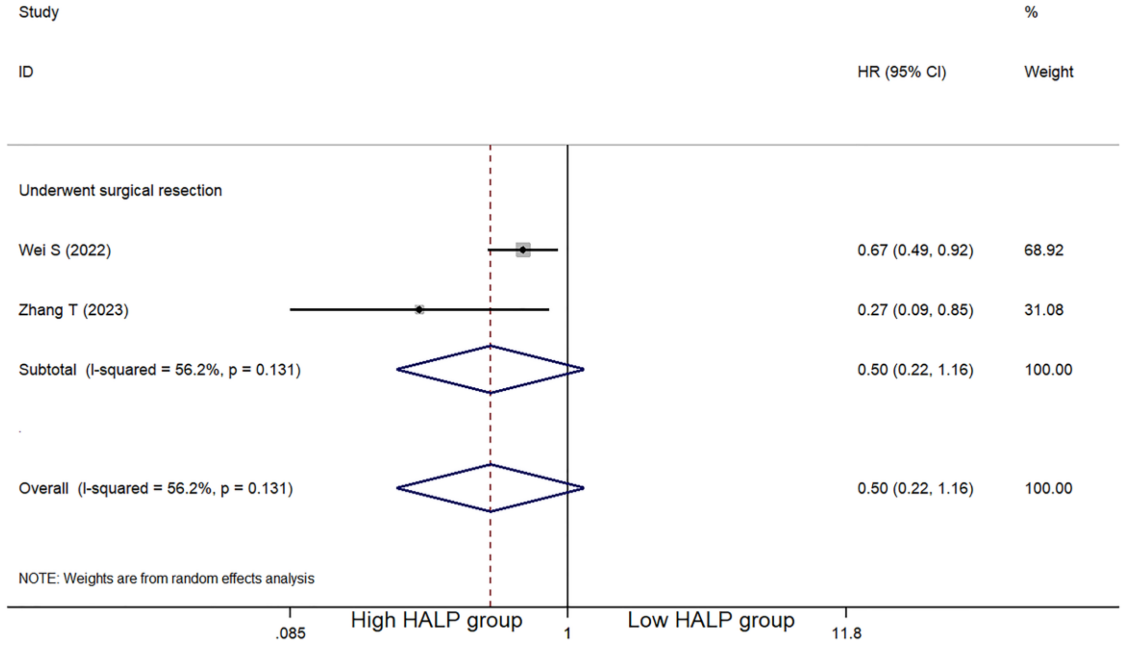

Figure S3: Multivariate analysis of the association between HALP score and disease-free survival in lung cancer patients.

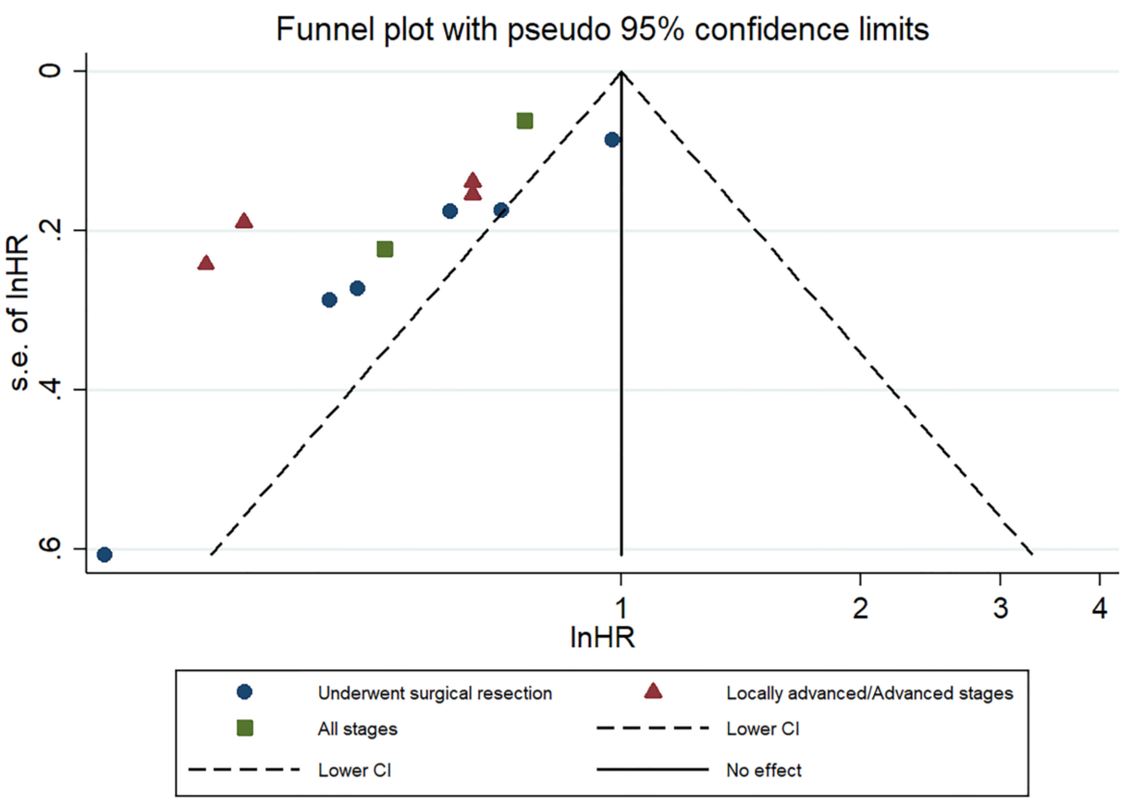

Figure S4: Funnel plot analysis for the association between HALP score and overall survival in lung cancer patients.
